# Searching for a compromise between biological and economic demands to protect vulnerable habitats

**DOI:** 10.1038/s41598-018-26130-z

**Published:** 2018-05-17

**Authors:** M. Grazia Pennino, Marie-Christine Rufener, Mario J. F. Thomé-Souza, Adriana R. Carvalho, Priscila F. M. Lopes, U. Rashid Sumaila

**Affiliations:** 1 Fishing Ecology Management and Economics (FEME) - Universidade Federal do Rio Grande do Norte – UFRN. Depto. de Ecologia, Natal (RN), Brazil; 2 Statistical Modeling Ecology Group (SMEG). Departament d’Estadística i Investigació Operativa, Universitat de València, C/Dr. Moliner 50, Burjassot, 46100 Valencia, Spain; 3 Instituto Español de Oceanografía. Centro Oceanográfico de Murcia. C/ Varadero 1, San Pedro del Pinatar, 30740 Murcia, Spain; 4 Universidade Federal de Sergipe – UFS. Depto. de Engenharia de Pesca e Aquicultura – DEPAq, São Cristóvão (SE), Brazil; 5 Fisheries Economics Research Unit, University of British Columbia, 2202 Main Mall, Vancouver BC, V6T 1Z4 Canada; 6 Technical University of Denmark, Institute for Aquatic Resources, Kemitorvet, Building 201, 2800 Kgs. Lyngby, Denmark

## Abstract

Identifying vulnerable habitats is necessary to designing and prioritizing efficient marine protected areas (MPAs) to sustain the renewal of living marine resources. However, vulnerable habitats rarely become MPAs due to conflicting interests such as fishing. We propose a spatial framework to help researchers and managers determine optimal conservation areas in a multi-species fishery, while also considering the economic relevance these species may have in a given society, even in data poor situations. We first set different ecological criteria (i.e. species resilience, vulnerability and trophic level) to identify optimal areas for conservation and restoration efforts, which was based on a traditional conservationist approach. We then identified the most economically relevant sites, where the bulk of fishery profits come from. We overlapped the ecologically and economically relevant areas using different thresholds. By ranking the level of overlap between the sites, representing different levels of conflicts between traditional conservation and fishing interests, we suggest alternatives that could increase fishers’ acceptance of protected areas. The introduction of some flexibility in the way conservation targets are established could contribute to reaching a middle ground where biological concerns are integrated with economic demands from the fishing sector.

## Introduction

Vulnerable habitats define critical areas for species, as the ones where they spawn, breed, feed, and/or grow to maturity. Effectively protecting them is an important demand that conservationists have in order to help restore sustainable exploitation of marine fishes^1–3^. However, identifying specific vulnerable areas is challenging because the habitat requirements of most species are still poorly understood^4^.

To date most approaches in marine conservation are based on identifying the habitat requirements of one species at a time, which is known as the single-species approach. This has resulted in extremely large areas being recommended for protection, which is usually difficult to implement in most contexts^5^, especially given the social and economic relevance of fishing. Therefore, a compromise needs to be sought in order to identify solutions that minimize both socioeconomic losses and the total amount of habitat needed for a given level of protection, while still encompassing as many species as possible.

To that end, rather than allocating vulnerable habitats on a species-specific basis, a possible solution could be to establish some criteria that encompass a variety of species and then determine what combinations of sites meet those criteria for all species. Obviously, depending on the criteria used, solutions will likely include a range of different species habitats with varying sizes for the areas to be protected.

One such criterion could be the simple identification of the main habitats used by a single species of ecological interest. However, this could result in inadequate choices, because such a broad approach makes it impossible to prioritize habitats for conservation and management^6^. A likely better alternative for choosing areas to be protected is to spatially identify ecological and/or biological criteria that could help replenish a variety of relevant species simultaneously.

However, vulnerable habitats based on one or multiple species rarely become protected areas or achieve any sort of conservation status mostly due to conflicting interests with human activities, especially fishing^7^. Such conflicts are not always driven by large companies’ profits and short-term gains, because fisheries also feed two and a half billion people around the world and provide over 200–260 million jobs directly and indirectly^8^. Therefore, fisheries are an important aspect of food security and livelihood maintenance, especially in developing tropical countries. In these countries, the activity is mainly carried out by artisanal fishers that use simple technologies and have little autonomy to reach farther fishing grounds, although this by no means exempts them from negatively impacting stocks^9^. Hence, given both the economic importance of fisheries and their negative ecological impact, the best conservation approach would be a compromise between ecological and economic goals^10^.

Several spatial zoning approaches that try to simultaneously ensure biodiversity and minimize negative impacts on stakeholders have been proposed^11–16^. However, these approaches generally rely on “data-rich” fisheries, whereas in developing countries data tend to be poorly documented and inadequately managed due to lack of research funding for monitoring and analysis^17^. Conventional analytical tools that require well-informed and detailed datasets (*e*.*g*. Zonation and Marxan) are not applicable in “data-poor” fisheries and hence reliable statistical tools that perform well with limited information still need to be developed^18,19^.

In this study, we propose a framework suited for data-poor situations to help researchers and managers decide on optimal conservation areas for intensively fished species that take into account the economic relevance such species have for a given society. To do so, we used three different ecological criteria (species resilience, vulnerability and trophic level) to identify vulnerable habitats for fisheries target species using data from multi-specific artisanal fisheries in Brazil. We then identified the most profitable areas and overlapped these with the ecologically optimal sites using different thresholds. Ultimately, we computed fishers’ economic losses by simulating the implementation of spatial closures in the identified areas. We assumed that the acceptance of a protected area would vary depending on the degree of overlap and possible economic losses for fishers. Therefore, conservation measures could be better tailored to increase acceptance and to accommodate different levels of compromise, where biological concerns are integrated with economic demands.

The proposed framework could be a powerful approach, even in data-poor fisheries, for reconciling the globally available environmental data with Bayesian models capable of dealing with limited information while simultaneously accounting for different sources of uncertainty^19^. Unlike other spatial zoning tools, the proposed decision-supporting mechanism could be considered a flexible white-box approach and be easily adapted to both terrestrial and aquatic contexts.

## Materials and Methods

### The spatial-economic framework in five steps

**Figure 1:**
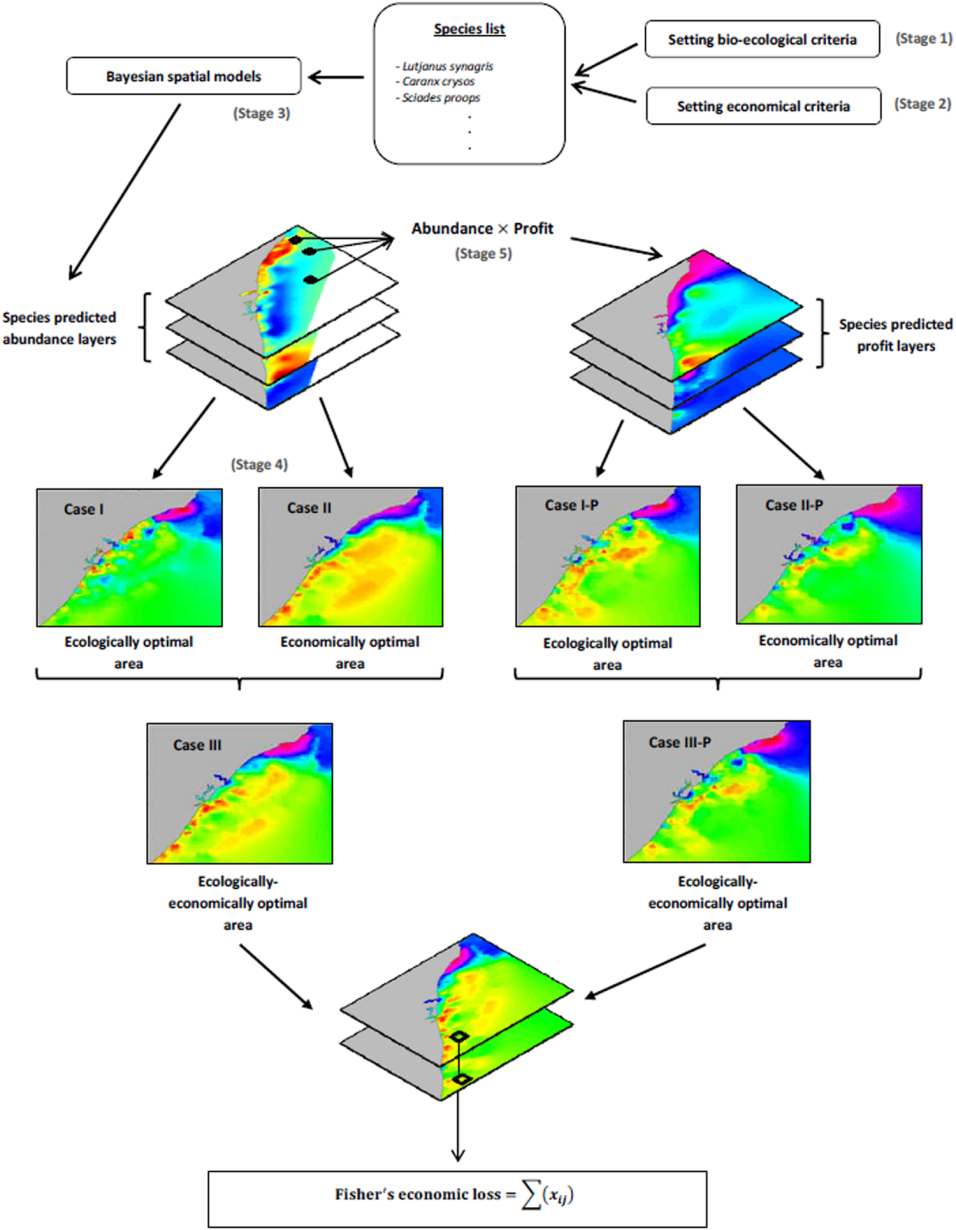
Diagram of the five steps of the analytical procedure adopted. For fisher’s economic loss x_ij_ denotes the profit for each longitude i and latitude j comprised between the 90% percentile and the maximum value.

The analytical strategy we adopted involved five steps resumed in Fig. 1: (1) setting a group of ecological/biological criteria to identify ecological optimal areas; (2) setting an economic criterion to identify economically optimal areas; (3) using Bayesian models to identify and predict species distribution; (4) overlapping the ecologically and economically optimal areas to identify the possible levels of conflict between conservation and economic interests using different thresholds, and (5) computing fishers’ economic losses for each proposed scenario assuming that the ecologically optimal area will be closed to fishing.

### The case study

To test our approach, we used data from the artisanal fisheries that took place on the continental shelves of two Brazilian states, Sergipe and the northernmost coast of Bahia, along approximately 240 km of coastline. In this area, as for most of the northeastern Brazilian states, small-scale fisheries harvest up to 90% of all the fish, being one of the main economic activities in the region^20^.

Harbor observers carried out the data sampling over three years (2012–2014) at thirteen different landing sites, distributed along the area. Information on fishing operations (date, landing site, name of the fishing ground) and vessel category (type, gear, and number of fishers) was obtained directly from the fishers. Fishing grounds were located by analyzing fishers’ GPS tracks, taking into account the speed and distance from the coast^21^. Specifically, we analyzed two different vessel categories that operate in this area: open water canoes (hereafter “OWC”) and motorized boats (“MB”). The first category included motorized sailing boats without a cabin (<10 m long). The second group included motorized vessels with cabins (10–15 m long). All the vessels considered in these two categories operated from the coastline to the shelf break up to 60 m deep.

Whenever possible, catches were identified to species level, otherwise to genus or family level. In total, we identified 84 species, but we excluded from our analyses those considered rare (<1% of the landings), which resulted in a final list of 26 species (Supplementary Tables S1 and S2).

#### Setting ecological criteria to identify ecologically optimal areas

We used three different ecological indexes to identify the ecologically optimal areas, namely: species vulnerability, resilience, and trophic level. These criteria were extracted from FishBase^22^ for all the species fished in the area that represented more than 1% of the catch (Table 1).

**Table 1:** Ecological and economic categories of the criteria selected for the spatial-economic framework for the twenty-six species analyzed that are present in artisanal fisheries. Values in parentheses indicate the numerical value for each category evaluated.

Species	Vulnerability	Resilience	Trophic level	Profitability (R$/Kg)^*^
Ariid group	Moderate (50)	**Low**	Medium (3.7)	Low (3.58)
*Caranx crysos*	Low (34)	Medium	**High** (4.1)	Medium (5.59)
*Caranx hippos*	**High** (58)	Medium	Medium (3.6)	Low (1.41)
*Centropomus* spp.	Moderate (42)	Medium	**High** (4.1)	**High** (15.89)
*Conodon nobilis*	Low (27)	Medium	Medium (3.6)	Low (4.18)
*Cynoscion* spp.	**High** (49)	Medium	Medium (3.8)	**High** (10.70)
*Dasyatis* spp.	**Very High** (90)	**Low**	Medium (3.1)	Medium (5.29)
*Diapterus auratus*	Low (26)	High	Low (2.4)	Low(1.28)
*Larimus breviceps*	Low (25)	High	Medium (3.5)	Low (3.09)
*Litopenaeus schmitti*	Low (10)	High	Low (2)	**High** (10.83)
*Lutjanus analis*	**High** (55)	**Low**	Medium (3.9)	Medium (8.54)
*Lutjanus jocu*	**High** (66)	**Low**	**High** (4.4)	**High** (9.06)
*Lutjanus* spp.	Moderate (51)	Medium	Medium (3.8)	Medium (7.68)
*Lutjanus synagris*	Moderate (38)	Medium	Medium (3.8)	Low (4.04)
*Macrodon ancylodon*	Moderate (36)	Medium	Medium (3.9)	Medium (6.39)
*Micropogonias furnieri*	Low (31)	Medium	Medium (3.1)	Medium (6.25)
*Myctoperca bonaci*	**High** (63)	**Low**	**High** (4.3)	**High** (9.32)
*Ocyurus chrysurus*	**High** (65)	**Low**	**High** (4.0)	Medium (6.14)
Prawn group	Low (10)	High	Low (2)	Medium (7.34)
*Sciades proops*	Moderate (38)	High	**High** (4.4)	Medium(5.40)
*Scomberomorus brasiliensis*	**High** (67)	Medium	Medium (3.3)	Low (4.81)
Scombrid group	Moderate (45)	Medium	**High** (4.4)	Medium (7.16)
*Seriola dumerili*	Moderate (54)	Medium	**High** (4.5)	Medium (7.60)
Shark group	**High** (62)	**Low**	**High** (4.2)	Medium (8.43)
Tunas group	**High** (57)	Medium	**High** (4.4)	Medium (6.13)
*Xiphopenaeus kroyeri*	Low (10)	High	Low (2)	**High** (9.92)

^*^Values are shown in Brazilian currency (BRZ). The average dollar conversion rate for the period consisted in 1 USD = 2.5 BRZ at the time of sampling collection (http://www.bcb.gov.br/txcambio).

The vulnerability index integrates the ecological characteristics of a species with its life history (maximum size at first maturity, age, longevity, growth parameter K von Bertalanffy, natural mortality, fertility, energy spatial behavior, and geographic reach), using the “*Fuzzy Expert System”* software^23^. It is different than the vulnerability index provided by the International Union for Conservation of Nature (IUCN), which takes into account fish range size. Whereas the FishBase index seems to be less accurate than the IUCN, it is available for most species and not only to those well-studied, being especially recommended for macroecological studies that include multiple species^24^, such as the present study.

Briefly, FishBase vulnerability index combines intrinsic vulnerability derived from life-history traits to some threatening factor such as fishing^23^, because most fish stock responses to exploitation are reflected in their life history and ecological characteristics^25^. Fuzzy logic is then applied to estimate a vulnerability index as an alternative to deal with knowledge vagueness. For instance, whereas it is well known that large fish tend to be associated to a higher extinction rate, there is no clear-cut measure to say what a large and what a small fish is. Hence, the vulnerability index essentially describes the relationship between the ecological characteristics through the fuzzy membership function, where instead of classifying membership as true or false (for example, a given fish is or is not considered to be a large-sized species), it is classified along a truth gradient. In this way, based on probability theory the same fish would have the possibility of being classified as both medium and large size, but with different degrees of membership. Once the life-history traits are analyzed as input variables for each species, the outputs are generated on an arbitrary scale varying from 0 to 100 and are classified into four categories. Values from 1 to 35 indicate species with low vulnerability; 36 to 55 are considered moderate; 56 to 75 are those with high vulnerability, and values above 76 are species with very high vulnerability^23^.

Similarly, the resilience index also aggregates several biological features (parameters) of a species life history (growth parameter K von Bertalanffy, the intrinsic rate of population growth, fecundity, life span, and age of first maturity). The intrinsic rate of increase is the main parameter to determine resilience because it embodies most of the other components^26^. However, because the FishBase method to estimate the intrinsic rate of increase is not yet fully reliable, species are conservatively assigned to the lowest category of resilience for which any of the other available parameters fits. Resilience is expressed on a categorical scale that ranges from high to very low resilience values^27^ (see http://www.fishbase.us/manual/English/key%20facts.htm#resilience for more details). For our study we selected species life-history parameters from the available data in FishBase as defaults for biological features equations as no additional information was known a priori. However, all parameters presented a 95% confidence limit, derived from the standardized residuals^26^.

Finally, trophic levels define the position of a species within the food web. The species trophic level index varies from 1 to 5, where 1 includes primary producers and detritus and 5 apex predators^27^. In reality, most species present a fractional trophic level because most feed on more than one level. Thus, a trophic level of 5 is rarely, if ever, observed among marine mammals^27^. The trophic level (*TL*) of a consumer species *i* is usually defined based on stomach contents and stable isotope analyses, and is calculated according to the formula:

1 $${{\rm{TL}}}_{{\rm{i}}}={\rm{\Sigma }}({{\rm{TL}}}_{{\rm{j}}}\ast {{\rm{Dc}}}_{{\rm{ij}}})$$

where *TL*_*j*_ is the fractional trophic level of species *j* (prey), and *DC*_*ij*_ is the fraction of *j* in diet *i*^28^.

We obtained the TL values of the selected species from FishBase (see http://www.fishbase.org for more details). In order to group different categories of TL, we carried out a Cluster analyses (CA) using a Euclidean distance matrix and the Ward’s clustering method. The similarity dendrogram from CA showed three main groups (Supplementary Fig. S1). Values over 3.9 were considered to be high trophic levels; 3.9 to 3.1 were considered medium and lesser than 3.1 were considered to be low trophic levels. High trophic level species are those usually more subjected to fishing pressure and therefore in more urgent need of conservation^29^.

We considered a species to be ecologically important if it fit at least one of the ecological criteria in a critical or worrisome situation, *i*.*e*., species with high vulnerability, low resilience and/or high trophic level.

#### Setting a criterion to identify economically optimal areas

We used the annual profit generated from fishing to define the economically optimal areas. We opted for profit instead of fish price because the latter does not consider abundance or the costs associated with the catch of low abundant species. For instance, a pricey species may be rare and costly to catch, contributing little to the final profit.

To compute annual profits we first calculated the annual revenues generated from fishing as:

2 $${{\rm{R}}}_{{s}}={{\rm{L}}}_{{s}}\ast {{\rm{V}}}_{{s}}$$

where R is revenue from catching species *s*, L_*s*_ is landings of each species in kg and V_*s*_ is the ex-vessel price of each species per kg. Harbor observers collected the ex-vessel price of each species in all the sampling sites monthly. This was done by extracting both the average price for the entire time series, and the number of harbors for each selected species.

Thus, profit is given by:

3 $${{\rm{P}}}_{{s}}={{\rm{R}}}_{{s}}-{{\rm{Tc}}}_{{s}}$$

where R_*s*_ is revenue per species and TC is total cost of fishing gear per kg for species *s*. Total costs were obtained through direct interviews with fishers. For each species, we attributed the TC of the fishing gear most commonly used to catch it.

Finally, we categorized the species in classes of profit using the same CA analytical procedure as described in the previous section. The CA procedure classified each species into one of three categories: low (<R$5.29/Kg), medium (R$5.30/KG to R$8.54/Kg) and highly (R$9.06/Kg to R$15.89/Kg) profitable (Table 1).

#### Modeling species distributions

We used Hierarchical Bayesian spatial models (HBSMs) to estimate the vulnerable habitat of all the species that were selected by the ecological and economic criteria, meaning those that showed low resilience and high vulnerability, trophic level, or profitability. This resulted in vulnerable habitat estimates for 18 out of 26 species. The abundance index was defined as the catch-per-unit-effort (CPUE), calculated as the species total catch (Kg) divided by the fishing effort. Specifically, fishing effort was determined as the number of fishers multiplied by the number of fishing days.

The spatial abundance data of these species have three main characteristics: strong spatial dependence, high proportions of zero, and a distribution highly skewed to the right. Therefore, we used a Bayesian hurdle spatial model, which is suitable for such situations. These models are carried out in two stages; one that models the presence/absence of the species using a binomial distribution, and the other that models the abundance with a gamma distribution. The use of a gamma distribution for species abundance proved to be effective to analyze skewed positive data^30^. Finally, a spatial random effect was included in both stages to account for spatial autocorrelation.

In addition to the spatial effect, we considered seven environmental variables to be possible determinants of a species in a given habitat: Sea Surface Temperature (SST), Chlorophyll-a concentration (Chl-a), Sea Surface Salinity (SSS), depth, slope and rugosity of the seabed, and distance to the coast (*see* Supplementary Information). These are variables that typically account for the occurrence of a species on a given site.

We used Vague Gaussian prior distributions (β ~ N (0,0.01)) for all the involved fixed parameters, while a prior Gaussian distribution with a zero-mean and covariance matrix of dependent hyperparameter was used for the spatial effect (see Muñoz^31^ for more detailed information about spatial effects). We carried out the Bayesian models using the *Integrated Nested Laplace Approximation* (INLA) methodology^32^ and package (http://www.r-inla.org) implemented in the R software^33^.

We used the Deviance Information Criterion (DIC)^34^ and the cross-validated logarithmic score (LCPO) measure^35^ to manually select the variables, based on a forward stepwise method. DIC measured the goodness of fit while LCPO measured the predictive quality of the models. Both measures are inversely related to the compromise between fit, parsimony, and predictive quality.

#### Overlapping the ecologically and economically optimal areas using different thresholds

The predicted abundance of highly vulnerable, high trophic level and low resilience species were overlapped in order to identify the ecological optimal areas. Similarly, the economically optimal area was defined by overlapping the predicted abundance of the species belonging to the highly profitable category.

As there is still no agreement in a quantitative threshold to define the best area for conservation purposes^36^, we evaluated three different thresholds to define the boundaries of the two optimal areas and the overlap between both optimal areas:

- Scenario 1: threshold bounds those locations containing the probability of 25–50% that the estimated species abundance falls within the identified area;
- Scenario 2: threshold bounds those locations containing the probability of 50–75% that the estimated species abundance falls within the identified area;
- Scenario 3: threshold bounds locations containing the probability of >75% that the estimated species abundance falls within the identified area.

For each scenario, the identified ecological and economically optimal areas were overlapped to assess possible levels of conflict between conservation and economic interests (Fig. 1).

Optimal areas were then compared using the Schoener’s and Warren’s similarity statistics^37^. These statistics range from 0, which indicates no overlap between areas, to 1, which means that the areas are identical. Both statistics assume probability distributions defined over geographic space, in which p_Xi_ (or p_Yi_) denotes the probability assigned by the model for the area X (or Y) to location *i*. Specifically, Schoener’s statistic for niche overlap is:

4 $${\rm{D}}({{\rm{p}}}_{{\rm{X}}},{{\rm{p}}}_{{\rm{Y}}})=1-1/2{\sum }_{{\rm{i}}}|{{\rm{p}}}_{{\rm{Xi}}}-{{\rm{p}}}_{{\rm{Yi}}}|$$

while Warren’s statistic is:

5 $${\rm{I}}({{\rm{p}}}_{{\rm{X}}},{{\rm{p}}}_{{\rm{Y}}})=1-1/2\,{{\rm{H}}}^{2}({{\rm{p}}}_{{\rm{X}}}-{{\rm{p}}}_{{\rm{Y}}})$$

which is based on the Hellinger distance, defined as:

6 $${\rm{H}}({{\rm{p}}}_{{\rm{X}}},{{\rm{p}}}_{{\rm{Y}}})=\surd {\sum }_{{\rm{i}}}{(\surd {{\rm{p}}}_{{\rm{Xi}}}-\surd {{\rm{p}}}_{{\rm{Yi}}})}^{2}$$

In order to apply this statistics, predictions were standardized between 0 and 1. These analyses were carried out using the *nicheOverlap* function of the *dismo* package^38^ in R software.

#### Computing fishers’ economic losses

Based on the optimal areas identified by the three different threshold outlined in the previous section, different trade-off scenarios were simulated to compute fishers’ economic losses.

For each scenario, profit maps were generated for all the modeled species found in the selected optimal areas and in the overlapped area. Provided that some fishing grounds may contain numerous individuals of a particular species with low economic importance and few individuals of another species with high economic importance, profit maps were computed as a weighted average between the species abundance and their profit. For each scenario fishers’ economic losses are then represented by the estimated profit values that would be obtained from the respective selected areas, in the event that some of these areas were closed to fishing (See stage 4 and 5 of Fig. 1).

### Data availability

The fishery dataset analyzed during the current study is included in the Supplementary Information files.

## Results

### Ecological and economic species list

Among the 26 species usually fished in the study area, eighteen species fitted at least one of the ecological or economic criteria. Of these species, sixteen fitted the ecological criteria and were therefore used to define ecological optimal areas, while six species were used to define the economic optimal areas (Table 1). Four species (*Cynoscion* spp., *Lutjanus jocu*, *Centropomus* spp., *Mycteroperca bonaci*) fitted both the ecological and economic criteria (Table 1).

### Vulnerable habitats for ecological and economic species

We fitted a total of eighteen Bayesian spatial models, corresponding to the eighteen species selected using the ecological or economic criteria (Table 1). All environmental predictors affected species abundance, except for the seabed and slope variables (Supplementary Table S3). The spatial distribution of the considered species was mainly driven by SSS, Chl-*a* concentration and distance to the coast (Supplementary Table S3). Overall, species abundance increased in areas with higher Chl-*a* concentrations and lower SSS values (Supplementary Table S3). Contrarily, *Caranx crysos* showed a negative relationship with Chl-*a* concentration (Supplementary Table S3). The rugosity was also a relevant factor for some species, highlighting the preference that some species, such as *Centropomus* spp. and Shark group, have for flat bottoms, while *Lutjanus analis* and *L*. *jocu* preferred more complex seabeds. The SST was relevant only for *Cynoscion* spp., whose abundance increased towards warmer waters (Supplementary Table S3). None of the environmental variables used were able to explain the abundance of *Xiphopenaeus kroyeri*, and the best selected model included only the spatial component as a relevant predictor (Supplementary Table S3).

Lastly, while some species clearly presented preference for deeper areas (e.g., *C*. *crysos*, *L*. *analis*, *L*. *jocu*, *M*. *bonaci*, *Ocyurus chrysurus*, *Scombrid group*, *Seriola dumerili* and *Tunas group*), most were more abundant in shallow waters (Supplementary Figure S2).

### Ecological and economic optimal areas

#### Scenario 1: Less conservative

**Figure 2:**
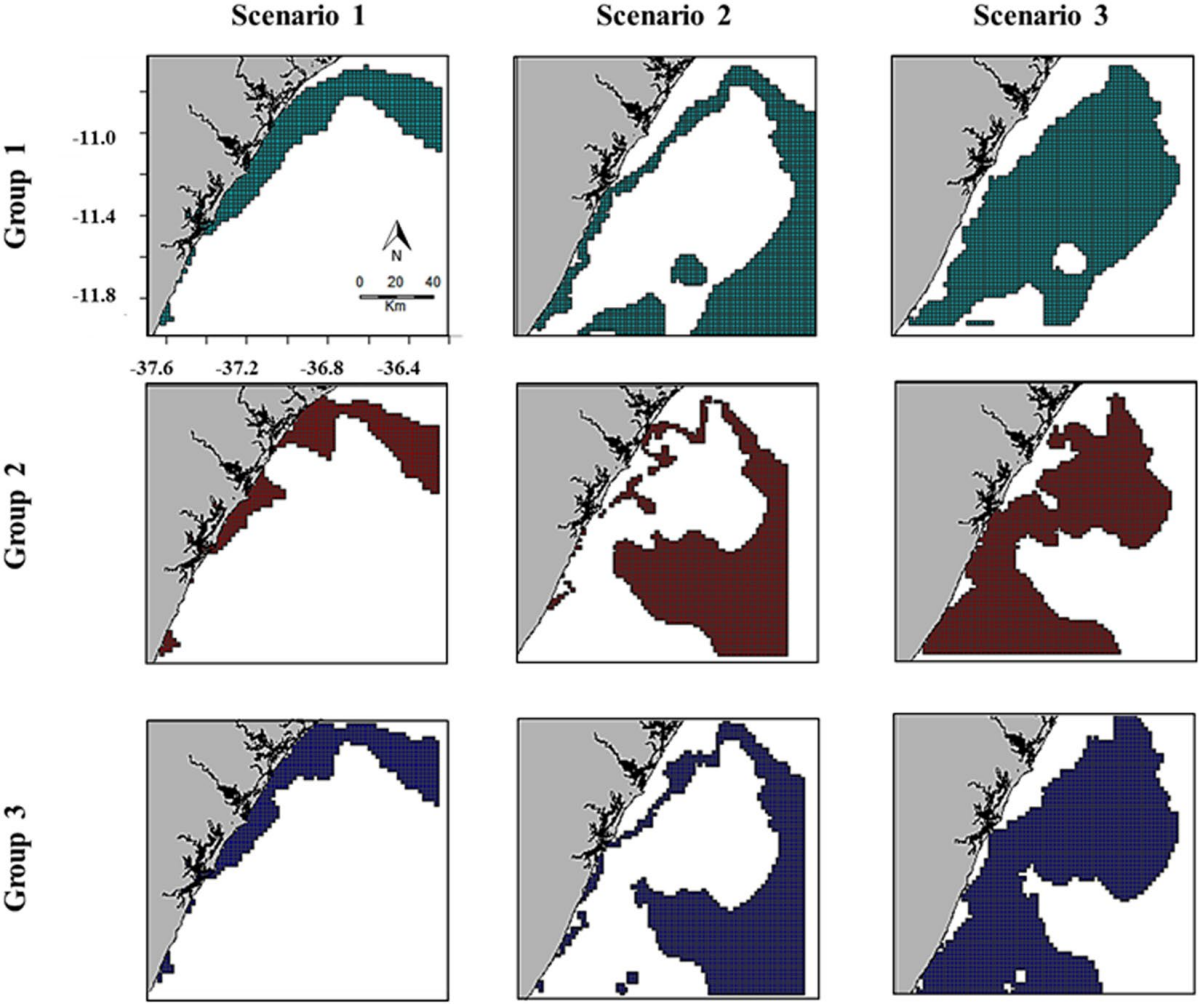
Maps showing the ecological (group 1), economic (group 2) and overlapped (group 3) optimal areas for the three different simulated scenarios.

In this scenario the ecological optimal area (3043.64 Km^2^) is larger than the economic optimal one (2559.12 Km^2^). These two areas are located in shallow waters where most small-scale fisheries operate and greatly overlap (I = 0.98; D = 0.87) (Fig. 2). The overlapped area includes the majority of the two optimal areas with a total extension of 2059.11 Km^2^, which corresponds to 58.2% of the total fishing area (Table 2).

**Table 2:** Numerical summary of the estimated profit and area extension for each considered group (ecological, economic and overlap between ecological and economic criteria) in each simulated scenario.

Groups	Scenario I		Scenario II		Scenario III	
	Profit (R$)	Area (Km^2^)	Profit (R$)	Area (Km^2^)	Profit (R$)	Area (Km^2^)
Ecological	2300.96	3043.64	8673.73	7867.98	13844.47	11213.13
Economic	4405.02	2559.12	14166.44	9016.37	15244.25	10313.52
Overlap	2567.23	2059.11	7134.75	5333.43	10713.57	7936.44
Total^*^	4138.7	3543.65	15705.38	11522.57	18375.20	13590.21

^*^${\rm{n}}({\rm{Ecologic}}\cup {\rm{Economic}})={\rm{n}}({\rm{Ecologic}})+{\rm{n}}({\rm{Economic}})-{\rm{n}}({\rm{Ecologic}}\cap {\rm{Economic}})$ – following the set theory.

From an economic point of view, if the ecological area were entirely closed to fishing, fishers would lose 85.9% of their fishing grounds (3043 Km^2^). This would correspond to a loss of 55.6% (R$ 2300) of their total annual profit. However, if only the ecological area that does not overlap with the economic one were closed to fishing, fishers would lose 27.78% of their fishing grounds (985 Km^2^) and have a lost of around 2% in their annual profit.

#### Scenario 2: Mildly conservative

Contrary to the scenario 1, in this situation the economic optimal area (9016.37 Km^2^) is larger than the ecological optimal one (7867.98 Km^2^). Here, the majority of the optimal areas are located in deeper waters, although some locations are closer to the coast (Fig. 2). Similarity statistics highlight a reasonable level of overlapping between the two areas (I = 0.85; D = 0.76). The estimated overlap area was 5333.43 Km^2^, which corresponds to 46.3% of the total fishing area (Table 2).

If the ecological area were entirely closed to fishing, fishers would lose 68% of their fishing grounds (7839 Km^2^). This would correspond to a loss of 55.2% (R$ 8673) of their total annual profit. Nevertheless, if only the ecological area that does not overlap with the economic one were closed to fishing, fishers would lose 21.8% of their fishing grounds (2506 Km^2^), representing a loss of R$ 1538 of their total annual profit (9.8%).

#### Scenario 3: Highly conservative

In this scenario the ecological optimal area (11213.13 Km^2^) is slightly larger than the economic optimal one (10313.52 Km^2^) (Fig. 2). Unlike the economic optimal area, the ecological optimal area is located in deeper waters, which are harder to reach by most small-scale fisheries. This scenario recorded the lowest similarity statistics between both optimal areas (I = 0.85; D = 0.73) where the overlapped area had an extension of 7936.44 Km^2^, which corresponds to 58.4% of the total area (Table 2).

Here, if the ecological area were entirely closed to fishing, fishers would lose 82.5% of their fishing grounds (11213 Km^2^), corresponding to a loss of 75.3% (R$ 13844) of their total annual profit. Notwithstanding, if only the ecological area that does not overlap with the economic one were closed to fishing, fishers would lose 24.1% of their fishing grounds (3276 Km^2^), which represents a loss of R$ 3130 of their total annual profit (17%).

## Discussion

We propose a spatial decision tool framework that takes into account both a diverse set of ecological criteria to identify vulnerable habitats for multiple species simultaneously and the economic relevance that these species have to fishers. Identifying such areas is a first step toward developing more agreeable sustainable fisheries management plans that protect marine resources while also providing the means for local livelihoods. Specifically, under the current used criteria, the designed vulnerable habitats offered a reasonable compromise between ecological and economic goals. Such compromises may be harder to reach when species vulnerability and economic subsidies that promote overfishing (*e*.*g*.: oil subsidy) are higher^39,40^. However, given that fish depletion may severely decrease human well-being^41^, and that rebuilding fish stocks can be costly in the short term^42^, middle grounds between conservation goals and economic exploitation could still be the best option, even under bleak perspectives, such as when fisheries are highly subsidized.

The results of our simulated trade-offs showed partial convergence between ecologically and economically optimal areas in all scenarios. About 50% of the total ecologically optimal areas overlapped with the economically optimal areas in all cases. This was expected as the most valuable species are often the ones at higher trophic levels, which usually means they also show low resilience and high vulnerability.

Among all the different scenarios, the third one was the most reliable from a conservationist point of view as it would preserve areas in which the probability of species abundance is higher than 75%. However, if the ecological area were entirely closed to fishing, it would be rated as the worst from an economic perspective due to the highest economic losses for the fishers.

Studies have shown that protected areas may need to cover at least 20–30% of the area of concern to be an effective tool in fisheries management^43^. In the case of the scenario 3, the higly conservative, if only the ecological area that does not overlap with the economic one were closed to fishing, approximately 41.4% of the ecological area could be completely protected, whereas 58.6% would be only partially protected with an economic loss for each fisher of almost 17% of their annual profit. In this case, even though it was the worst scenario with respect to economic losses, a reasonable trade-off between economic and ecological demands could still be reached. Scenario 2, the middly conservative one, represented the best compromise between ecological and economic benefits as it implied in the lowest economic losses while still encompassed areas of high species abundance. However, the identified vulnerable habitats of this scenario are predominantly located in deep waters, where some vulnerable species, such as *Centropomus* spp, will not be found. Therefore, this presents an expected trade-off when balancing ecological and economic goals, i.e., the area proposed to be protected will hardly ever be able to cover all the conservation aspirations, and some vulnerable species might be left behind such as *Centropomus* spp. Conversely, scenario 1, the less conservative, is the only one that preserves shallow waters where some of the most vulnerable species are abundant (e.g. *Scomberomorus brasiliensis*, *C*. *hippos*, Shark group and *Dasyatis spp*.). Yet, this scenario recorded the worst trade-offs among ecological and economic demands, as it is the one containing the lowest probabilities of species’ abundance and also one presenting the highest losses for fishers (~55% annual profits).

Under varying degrees of overlap, different management options could be proposed to reconcile conservation measures with human uses. For example, spatial-temporal closures of the ecological optimal area could be proposed. Partial protection for the overlapped area could be implemented by: (i) allowing fishing for only a limited number of vessels and for a restricted period of time during the year (*e*.g.: during the non-reproductive period of the most vulnerable species); or (ii) by splitting the overlapped area into two sub-areas for pulse fishing during alternate periods. That is, while fishing is allowed in one area, the other is let alone to recover^44^. However, even though harvesting closures are a common measure within contemporary community-based and co-management frameworks, managers have to consider that periodic closures may confer fisheries benefits for some taxa or in certain conditions but its efficiency for the management of all types of fisheries remains uncertain^45^.

Another perspective to be considered is that areas of both ecological and economic relevance could be more easily managed because they require minimal additional community engagement for success^46^. In these cases the community could be directly interested in protecting the resources because one could easily quantify the economic value of the conservation measures. Indeed, a two-way engagement process could be achieved through adaptive co-management and social-learning processes that engage both scientists and local community members^47^. Key ingredients to this approach should include collaboration with stakeholders, mainstreaming of scientific information (*e*.g., enhanced communication with communities through maps, scenarios, etc.), education and awareness-raising strategies to inform local communities about ecological values that they do not currently recognize, and incentive programs (*e*.*g*., grants, payments for ecosystem services, auctions for conservation contracts) to further support conservation actions.

It is noteworthy, however, that in all cases community and institutional capacities are determinant factors that need to be evaluated *a priori*^48^. Community capacity refers to the rules, procedures and values that people hold, which predispose them to work collectively for mutual benefit^44^. Institutional capacity is the ability of government agencies to provide public goods and services and ensure that laws and regulations will be enforced. Several methods have been proposed to compute community and institutional capacities^49^. A feasible tool could be to carry out interviews with the local community to identify the degree of acceptance of a possible spatial-temporal closure of the ecological optimal area (community capacity), and look at how much the government budget is dedicated to conservation measures (institutional capacity).

Traditionally, conservation planning is based on a systematic and scientific assessment of patterns that take into account only ecological criteria. Such proposals have become more accurate over time, and in areas where they have been effectively implemented they show positive signs of species protection and recovery. However, in countries where enforcement is lax and conservation *per se* can be a short-term burden to the poor, unless associated to compensatory mechanisms, they tend to not succeed^41^. Therefore, the proposed approach could minimize the conflicting goals between economic and ecological interests. This can be achieved firstly by minimizing the vulnerable habitat proposed to be protected, due to the multi-species approach, and secondly by identifying areas where some compromise would need to be made.

Lastly, it should be stressed that none of our presented models accounted for ontogenetic shifts of the evaluated species^50^, a caveat attributed to the data. Without appropriate biometric data, we assumed that the resultant spatial distribution ranges accounted relatively well for the species occurrence throughout their different life stages. However, it is known that species usually have different habitat requirements along their life history. For example, most species have different nursery areas than those where they will occur later in their development due to changes in their resource requirements^51^. However, our framework accommodates the incorporation of this type of information (*e*.*g*. competition, interaction, dispersal, and ontogenetic shift), whenever they are available, in a straightforward way. Basically, it would require the update of distribution maps to include, for instance, the ontogenetic movements of fisheries target species^19^.

The framework we presented here faces the challenge of combining actions that have been successful elsewhere with a new spatial-economic approach that uses novel techniques and available on-line data for any ecosystem and, especially, for data-poor situations. It is important to constantly refine and evaluate management measures as more information is acquired (on life history features, for instance) or as new situations emerge (e.g., climate change). Therefore, it is important to have such straightforward frameworks that could easily evolve into refined management alternatives.

When the usual conservation methods fail to reach compliance, new approaches may be key to find solutions that could be mutually beneficial to both conservation and fisheries, thus resulting in higher acceptance rates by fishers in the short-term, while still assuring that stocks will stand a chance to recover in the long run.

## Electronic supplementary material

Supplementary information
