# Supplementary material for: Searching for a compromise between biological and economic demands to protect vulnerable habitats
Source: Sci Rep. 2018 May 17;8:7791. doi: 10.1038/s41598-018-26130-z (PMC5958106; doi:10.1038/s41598-018-26130-z)
Supplement: Supplementary file 1 — Supplementary information [file 41598_2018_26130_MOESM1_ESM.doc]

**Searching for a compromise between biological and economic demands to protect vulnerable habitats**

M. Grazia Pennino^*^, Marie-Christine Rufener, Mario J. F. Thomé-Souza, Adriana R. Carvalho, Priscila F. M. Lopes, U. Rashid Sumaila

**Vessel categories datasets**

*Table S1: Main species caught by artisanal coastal fishers in the Brazilian northeast, using open water canoes (OWC).*

| **Family** | **Species** | **Total weight (kg)** | **Relative abundance (%)** |
| --- | --- | --- | --- |
| Sciaenidae | *Micropogonias furnieri* | 54794.90 | 19.58% |
| Sciaenidae | *Macrodon ancylodon* | 40297.22 | 14.40% |
| Ariidae | *Bagre* spp.*, Sciades* spp.  (Ariid group) | 30531.00 | 10.91% |
| Carcharhinidae | *Rhizoprionodon porosus, Carcharhinus limbatus*  (Shark group) | 25859.50 | 9.24% |
| Ariidae | *Sciades proops* | 24041.09 | 8.59% |
| Sciaenidae | *Cynoscion* spp. | 23472.92 | 8.39% |
| Dasyatidae | *Dasyatis* spp. | 16152.39 | 5.77% |
| Haemulidae | *Conodon nobilis* | 12801.71 | 4.57% |
| Lutjanidae | *Larimus breviceps* | 12498.70 | 4.47% |
| Centropomidae | *Centropomus* spp. | 6933.99 | 2.48% |
| Scombridae | *Scomberomorus brasiliensis* | 3112.29 | 2.34% |
| Carangidae | *Caranx hippos* | 3329.00 | 1.19% |
| Gerreidae | *Diapterus auratus* | 3112.29 | 1.11% |

*Table S2: Main species caught by artisanal coastal fishers in the Brazilian Northeast using motorized boats (MB).*

| **Family** | **Species** | **Total weight (kg)** | **Relative abundance (%)** |
| --- | --- | --- | --- |
| Penaeidae | *Xiphopenaeus kroyeri* | 143447.36 | 38.58% |
| Penaeidae | Prawn group | 21746.50 | 5.85% |
| Lutjanidae | *Lutjanus jocu* | 17937.50 | 4.82% |
| Lutjanidae | *Lutjanus* spp*.* | 16728.40 | 4.50% |
| Penaeidae | *Litopenaeus schmitti* | 14941.50 | 4.02% |
| Lutjanidae | *Lutjanus analis* | 13796.39 | 3.71% |
| Sciaenidae | *Macrodon ancylodon* | 13252.50 | 3.56% |
| Carangidae | *Caranx crysos* | 11528.50 | 3.10% |
| Serranidae | *Mycteroperca bonaci* | 10492.50 | 2.82% |
| Scombridae | *Acanthocybium solandri, Scomberomorus cavalla*  (Scombrid group) | 9065.50 | 2.44% |
| Carcharhinidae | *Rhizoprionodon porosus, Carcharhinus limbatus*  (Shark group) | 8703.50 | 2.34% |
| Carangidae | *Seriola dumerili*. | 8476.42 | 2.28% |
| Scombridae | *Thunnus spp., Katsuwonus pelamis, Auxis* spp*.*  (Tuna group) | 7073.50 | 1.90% |
| Carangidae | *Caranx hippos* | 5276.52 | 1.42% |
| Lutjanidae | *Ocyurus chrysurus* | 5188.45 | 1.40% |
| Sciaenidae | *Cynoscion* spp. | 5154.43 | 1.39% |
| Dasyatidae | *Dasyatis* spp. | 4738.50 | 1.27% |
| Ariidae | *Bagre* spp*., Sciades* spp.  (Ariid group) | 4429.45 | 1.19% |
| Lutjanidae | *Lutjanus synagris* | 3871.45 | 1.04% |


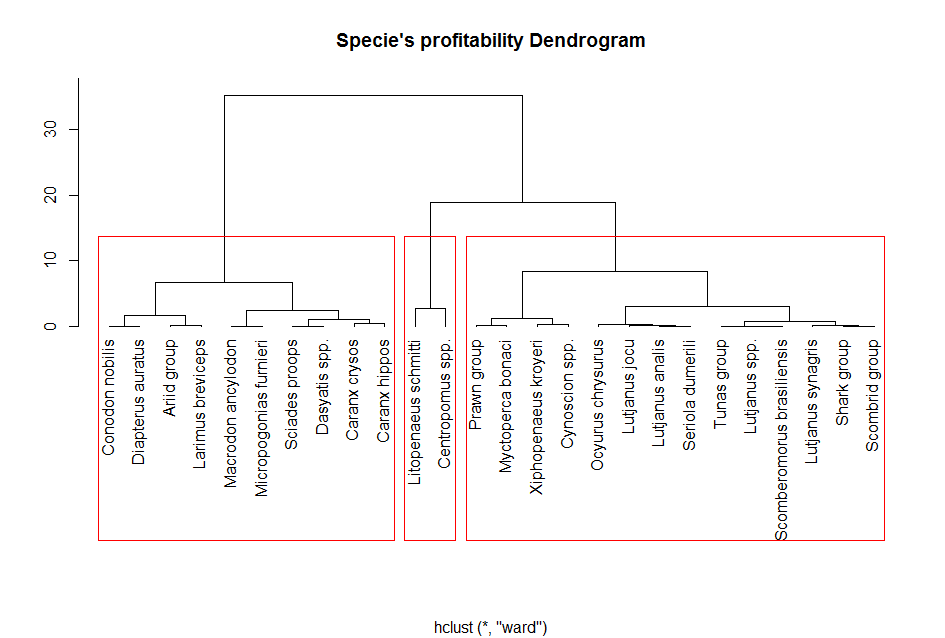

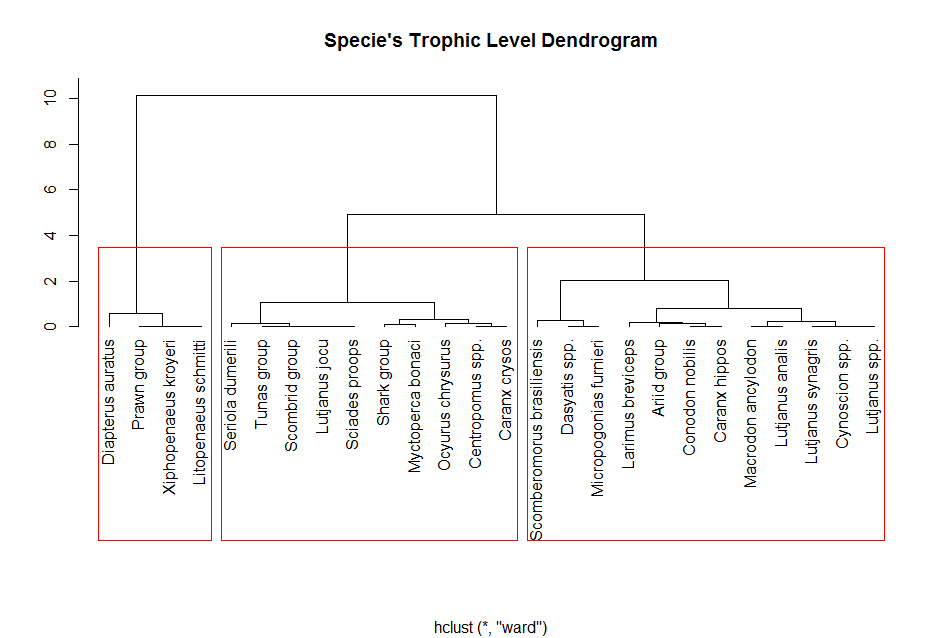


**A**

**B**

*Figure S1: Hierarchical cluster dendrogram for specie´s profitability (A) and trophic level (B) values.*

**Environmental data**

To evaluate species’ EFHs, we used seven predictors commonly used for marine species, which comprised sea surface temperature (SST, °C), depth (m), distance to coast (km), sea surface salinity (SSS, PSU), slope of the seabed (degree), chlorophyll-a concentration (Chl-a, mg/m^3^) and rugosity (index). Specifically, SST, SSS, depth, slope and distance to coast were extracted from the database provided by the ocean climate layers for marine spatial ecology (MARSPEC), which is available at [http://www.marspec.org](http://www.marspec.org/). Rugosity was derived from the depth layer by means of the Terrain Ruggedness Index^1^ provided from the *terrain analysis* plugin in Quantum GIS 2.8.7 software^2^. Chl-a concentration was extracted as nightly monthly means from the AQUA/MODIS satellite provided by the Nasa Earth Observations (NEO, at [http://www.neo.sci.gsfc.nasa.gov](http://www.neo.sci.gsfc.nasa.gov/)).

All environmental variables were aggregated at the same spatial resolution of 0.5°×0.5° and standardized in order to reduce correlation among model coefficients and to enable comparison of relative weights between variables. Multicollinearity was checked using Pearson’s (or Spearman’s, when necessary) correlation index. Since a high correlation was observed between Chl-a and distance to coast, as well as between depth and slope, we avoided to use them together during the modelling procedures.

Apart from the QGIS rugosity treatment, the remaining procedures were conducted using the R statistical software^3^ and the following packages: *raster*^4^, *rasterVis*^5^, *maptools*^6^, *gridExtra*^7^, *lattice*^8^ and *fields*^9^.

*Table S3: Numerical summary of the posterior distribution of the fixed effects for the best model of the selected species. This summary contains mean, the standard deviation (SD), and a 95% credible interval, which is a central interval containing 95% of the probability under the posterior distribution. (SST = Sea Surface Temperature; DC = Distance to Coast; SSS = Sea Surface Salinity; B = Bathymetry; Chl-a = Chlorophyll-a concentration; R = Rugosity).*

| Species | Predictors | Mean | SD | CI_95%_ | |
| --- | --- | --- | --- | --- | --- |
|  |  |  |  | Q_0.025_ | Q_0.975_ |
| Ariid group | Intercept | 0.1556 | 0.1768 | -0.2055 | 0.4944 |
|  | DC | -0.2454 | 0.0676 | -0.3772 | -0.1107 |
| *Caranx crysos* | Intercept_z | -8.7026 | 1.4427 | -12.499 | -6.4757 |
|  | Intercept_y | -6.965 | 2.0998 | -12.452 | -3.5716 |
|  | SSS | 1.879 | 0.324 | 1.2714 | 2.5494 |
|  | Chl-a | -1.9894 | 0.2202 | -2.4519 | -1.5857 |
| *Caranx hippos* | Intercept_z | -2.8611 | 0.4122 | -3.6979 | -2.0192 |
|  | Intercept_y | 0.9628 | 0.0746 | 0.8019 | 1.0986 |
|  | B | -0.0952 | 0.0291 | -0.1464 | -0.032 |
| *Centropomus* spp*.* | Intercept_z | -4.7851 | 0.9571 | -6.9858 | -3.1054 |
|  | Intercept_y | 1.2838 | 0.1125 | 1.0732 | 1.4986 |
|  | R | 0.1057 | 0.03 | 0.047 | 0.1649 |
| *Cynoscion* spp*.* | Intercept_z | -1.1604 | 0.504 | -2.2746 | -0.2022 |
|  | Intercept_y | 0.9865 | 0.2211 | 0.4924 | 1.4019 |
|  | SSS | -0.2204 | 0.048 | -0.3216 | -0.1338 |
|  | SST | 0.0928 | 0.036 | 0.0233 | 0.165 |
| *Dasyatis* spp*.* | Intercept_z | -2.2095 | 0.2061 | -2.6188 | -1.7992 |
|  | Intercept_y | 1.9254 | 0.0914 | 1.7403 | 2.1026 |
|  | B | -0.1237 | 0.0564 | -0.2366 | -0.0154 |
| *Litopenaeus schmitti* | Intercept_z | -9.6476 | 2.7648 | -15.369 | -4.4721 |
|  | Intercept_y | 0.9743 | 0.3984 | 0.0247 | 1.5244 |
|  | SSS | -0.1799 | 0.087 | -0.3457 | -0.0031 |
|  | DC | 0.5422 | 0.1047 | 0.3441 | 0.757 |
| *Lutjanus analis* | Intercept_z | -6.0399 | 1.6194 | -9.2691 | -2.8835 |
|  | Intercept_y | 0.0268 | 0.4768 | -0.9142 | 0.7643 |
|  | DC | 0.3195 | 0.0661 | 0.1806 | 0.4415 |
|  | R | -0.1997 | 0.0778 | -0.3615 | 0.0555 |
| *Lutjanus jocu* | Intercept_z | -5.586 | 3.381 | -12.352 | 0.9561 |
|  | Intercept_y | -0.0498 | 1.5358 | -3.1132 | 2.9342 |
|  | SSS | -0.878 | 0.1826 | -1.2542 | -0.5387 |
|  | Chl-a | 0.6445 | 0.1331 | 0.3982 | 0.6247 |
|  | R | -0.2928 | 0.088 | -0.4687 | -0.2899 |
| *Myteroperca bonaci* | Intercept_z | -6.1171 | 2.9137 | -11.906 | -0.4402 |
|  | Intercept_y | -1.1156 | 1.0584 | -3.2164 | 0.9481 |
|  | Chl-a | 0.4338 | 0.0911 | 0.2573 | 0.615 |
| *Ocyurus chrysurus* | Intercept_z | -8.8023 | 5.2462 | -19.173 | 1.446 |
|  | Intercept_y | -1.8085 | 2.1112 | -5.9789 | 2.3199 |
|  | Chl-a | 0.6546 | 0.1397 | 0.3881 | 0.9368 |
| *Sciades proops* | Intercept_z | -0.7358 | 0.3971 | -1.5381 | 0.1439 |
|  | Intercept_y | 1.2185 | 0.5955 | 0.0177 | 2.531 |
|  | SSS | -0.7092 | 0.113 | -0.9618 | -0.5127 |
|  | DC | -0.2167 | 0.1082 | -0.4377 | -0.0132 |
|  | B | -1.0106 | 0.2826 | -1.5874 | -0.4764 |
| *Scomberomorus brasiliensis* | Intercept_z | -3.4101 | 0.4334 | -4.3785 | -2.6595 |
|  | Intercept_y | 0.0539 | 0.2035 | -0.3867 | 0.4181 |
|  | SSS | 0.9077 | 0.5037 | 0.2744 | 2.188 |
| Scombrid group | Intercept_z | -3.8919 | 4.1117 | -12.035 | 4.2576 |
|  | Intercept_y | 0.1287 | 1.9322 | -3.7155 | 3.9837 |
|  | SSS | -0.9008 | 0.2134 | -1.3539 | -0.5083 |
|  | Chl-a | 0.4919 | 0.1499 | 0.222 | 0.8159 |
| *Seriola dumerili* | Intercept_z | -6.6 | 6.4776 | -19.426 | 6.0955 |
|  | Intercept_y | -0.4421 | 1.6905 | -3.8137 | 2.8552 |
|  | SSS | -0.4455 | 0.1195 | -0.6826 | -0.213 |
|  | DC | -0.1642 | 0.0728 | -0.311 | -0.0249 |
| Shark group | Intercept_z | -0.6255 | 0.2912 | -1.2 | -0.0352 |
|  | Intercept_y | 1.4523 | 0.1182 | 1.2198 | 1.693 |
|  | SSS | -0.3361 | 0.0414 | -0.4198 | -0.2574 |
|  | Chl-a | -0.1091 | 0.0342 | -0.1767 | -0.0421 |
|  | R | 0.0589 | 0.0284 | 0.0037 | 0.115 |
|  | B | -0.1148 | 0.0283 | -0.1676 | -0.0563 |
| Tunas group | Intercept_z | -4.8127 | 7.8491 | -20.25 | 10.6262 |
|  | Intercept_y | 1.3239 | 4.5506 | -7.6288 | 10.2768 |
|  | SSS | -2.2945 | 0.6105 | -3.4739 | -1.0775 |
|  | B | -0.2357 | 0.1022 | -0.4429 | -0.0412 |
| *Xiphopeaneus kroyeri* | Intercept_z | -5.3936 | 0.7682 | -7.1167 | -4.0658 |
|  | Intercept_y | 2.7026 | 0.3111 | 2.0065 | 3.239 |


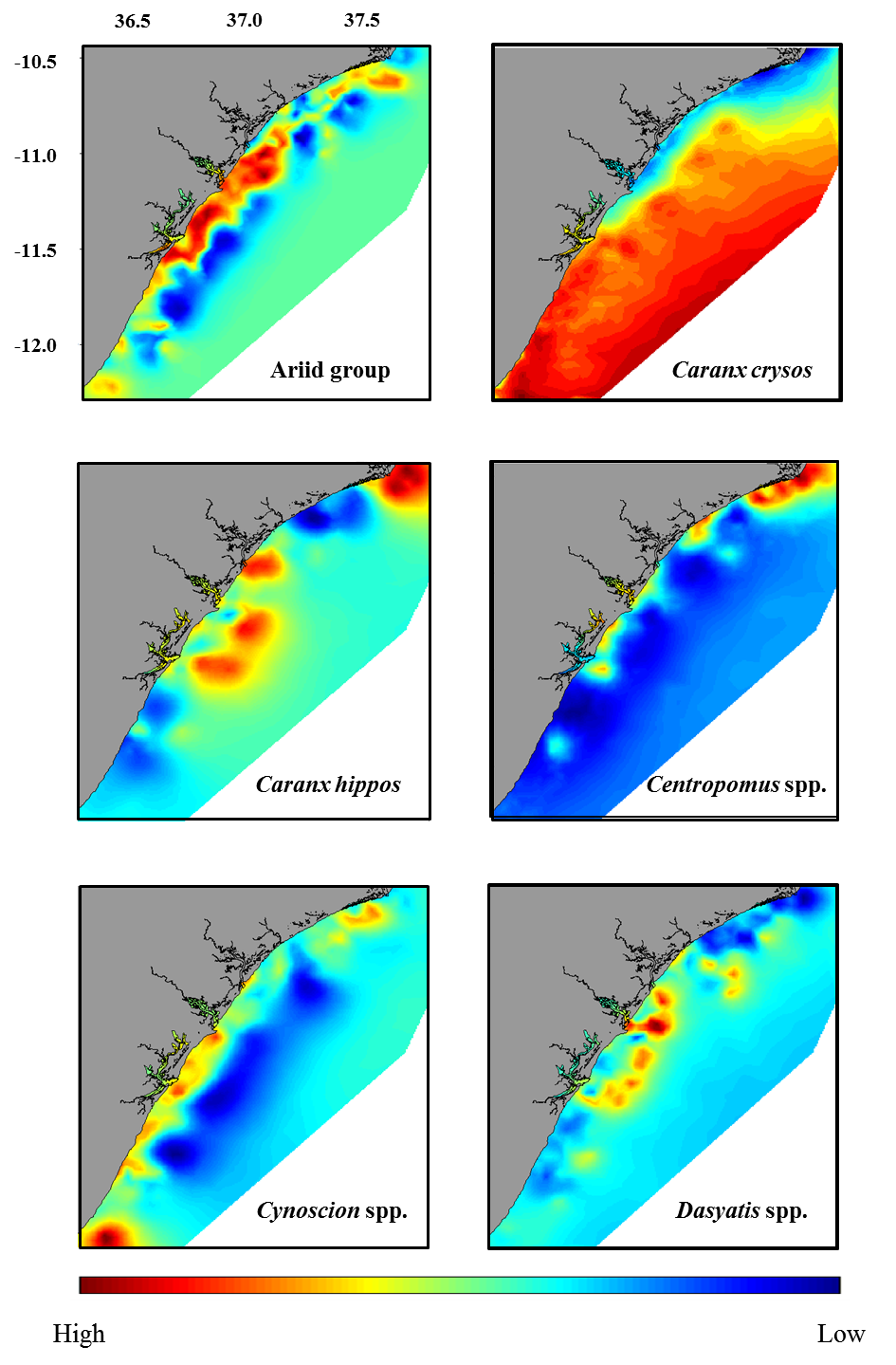


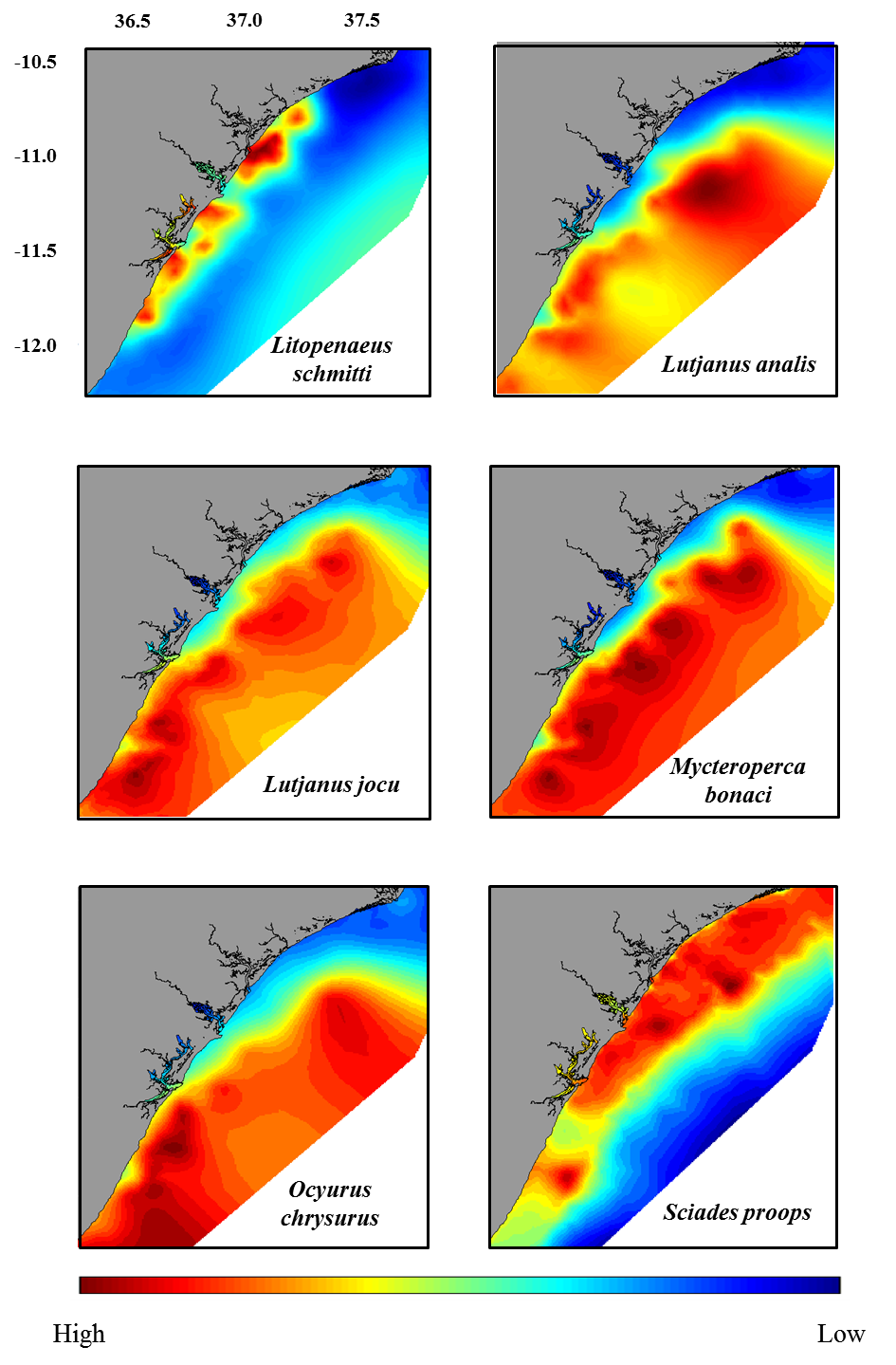


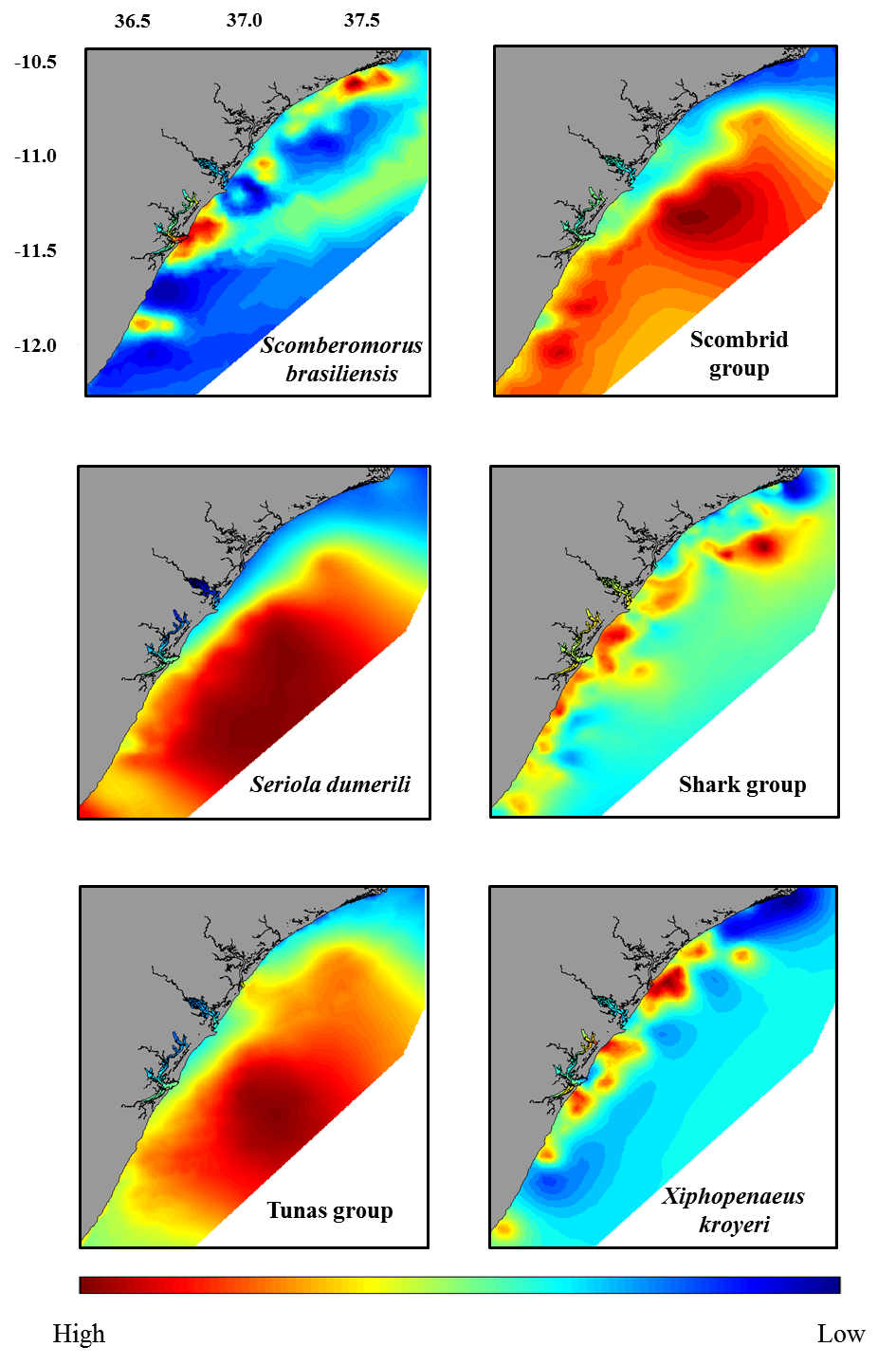


*Figure S2: Maps of the posterior predicted mean abundance of the selected species.*

**References**

1. Riley, S., DeGloria, S. & Elliot, R. A terrain ruggedness index that quantifies topographic heterogeneity. Intermt. J. Sci. (1999).

2. QGIS Development Team. QGIS Geographic Information System. Open Source Geospatial Foundation Project. (2017).

3. R Development Core Team. R: a language and environment for statistical computing. (2017).

4. Hijmans, R.J.. Raster: geographic data analysis and modeling. R package version 2.3-40. <http://CRAN.R-project.org/package=raster>/ (accessed 10.08.2015) (2015).

5. Lamigueiro, O.P. & Hijmans, R. RasterVis. R package version 0.31, doi: 10.5281/zenodo.12394. (2014).

6. Bivand, R. & Lwein-Koh, N. Maptools: tools for reading and handling spatial objects. R package version 0.8-34 <http://CRAN.R-project.org/package=maptools/> accessed (10.08.2015) (2015).

7. Auguie, B. GridExtra: functions in grid graphics. R package version 0.9.1, <http://CRAN.R-project.org/package=gridExtra/> (accessed 10.08.2015) (2012).

8. Deepayan, S. Lattice: multivariate data visualization with R, first ed. Springer, New York. (2008).

9. Nychka, D., Furrer, R. & Sain, S. Fields: tools for spatial data. R package version 8.2-1. <http://CRAN.R-project.org/package=fields/> (accessed 10.08.2015) (2015).
